# Silica and Silica–Titania Xerogels Doped with Iron(III) for Total Antioxidant Capacity Determination

**DOI:** 10.3390/ma14082019

**Published:** 2021-04-17

**Authors:** Maria A. Morosanova, Ksenia V. Chaikun, Elena I. Morosanova

**Affiliations:** 1 Analytical Chemistry Division, Chemistry Department, Lomonosov Moscow State University, 119234 Moscow, Russia; m.a.morosanova@gmail.com; 2 Department of Materials Science, Lomonosov Moscow State University, 119234 Moscow, Russia; conc.lab.student@gmail.com

**Keywords:** silica–titania xerogel, iron doped sol-gel materials, sensor materials, ferric reducing antioxidants power (FRAP), total antioxidant capacity determination, beverages analysis

## Abstract

In order to design a sensor material for total antioxidant capacity determination we have prepared silica and silica–titania xerogels doped with iron(III) and modified with 1,10-phenanthroline. Titanium(IV) tetraethoxyde content in the precursors (titanium(IV) tetraethoxyde and tetraethyl orthosilicate) mixtures has been varied from 0 to 12.5% vol. Iron(III) concentrations in sol has been varied from 1 to 100 mM. The increase of titanium(IV) content has led to a decrease in BET surface area and average pore diameter and an increase of micropore surface area and volume, which has resulted in better iron(III) retention in the xerogels. Iron(III), immobilized in the xerogel matrix, retains its ability to form complexes with 1,10-phenanthroline and to be reduced to iron(II). Static capacities for 1,10-phenanthroline have been determined for all the iron(III) doped xerogels (0.207 mmol/g–0.239 mmol/g) and they are not dependent on the iron(III) content. Sensor materials—xerogels doped with iron(III) and modified with 1,10-phenanthroline—have been used for antioxidants (catechol, gallic and ascorbic acids, and sulphite) solid phase spectrophotometric determination. Limits of detection for catechol, gallic and ascorbic acids, and sulphite equal 7.8 × 10^−6^ M, 5.4 × 10^−6^ M, 1.2 × 10^−5^ M, and 3.1 × 10^−4^ M, respectively. The increase of titanium(IV) content in sensor material has led to an increase of the reaction rate and the sensitivity of determination. Proposed sensor materials have been applied for total antioxidant capacity (in gallic acid equivalents) determination in soft beverages, have demonstrated high stability, and can be stored up to 6 months at room temperature.

## 1. Introduction

Antioxidants can be defined as substances that significantly delay or inhibit oxidation reactions. The consumption of antioxidants is an important step to fight against oxidative damage diseases and aging. Due to the large diversity of these compounds and their simultaneous presence in various samples, antioxidant concentrations are generally characterized as total antioxidant capacity (TAC) [1]. Gallic acid is generally employed as a reference standard for the detection of TAC [2].

Given the importance of TAC determination there is a great variety of electrochemical and spectroscopic procedures that are based on oxidation-reduction reactions where antioxidants act as reductants [3,4]. Spectroscopic TAC determination procedures use synthetic free radicals (ABTS, DPPH, etc.), transition metals complexes (iron (III), copper(II), and chromium(VI) [5,6,7,8,9,10,11,12,13,14]), metal ions (iron(III) and cerium(IV) [2,15]), and nanoparticles [16,17,18,19].

Procedures based on transition metal complexes offer some advantages: easily observed color change, fast reaction, and the possibility to adjust the standard potential with the selection of proper chromogenic ligand [6]. The procedures based on iron(III) and copper(II) complexes are the most widely used for TAC determination. The ferric-reducing antioxidant power (FRAP) assay uses iron(III) complexes with tripyridyltriazine (TPTZ) [5] or 1,10-phenanthroline (Phen) [6]. The cupric reducing antioxidant capacity (CUPRAC) assay uses copper(II)–neocuproine complex [7].

Modern development of FRAP and CUPRAC procedures includes the design of optical chemical sensors for simple, inexpensive, sensitive, and rapid TAC determination. Optical chemical sensors are miniaturized devices needed for applications such as biomedical research, environmental imaging, and industrial process control [7]. These sensors can be produced by immobilizing metal complexes onto appropriate solids, i.e., developing sensor materials. Such sensor materials are efficient for colorimetric and spectroscopic determinations. A few solid matrices have been employed up to date for FRAP reagent immobilization (paper [12], polymethacrylate [13], and Nafion membrane [14]) and in CUPRAC reagent immobilization (carrageenan film [7] and Nafion membrane [9,10]).

Silica and silica–titania sol-gel materials present a stable matrix with easily adjustable composition and properties for analytical reagents [20]. Silica–titania xerogels have been proposed as sensor materials for the determination of some antioxidants (ascorbic acid and polyphenols) based on the complex forming reactions with titanium(IV) [21]. The FRAP reagent has not been immobilized in sol-gel matrix yet. There are several works describing iron(III) immobilization [22,23,24,25,26,27,28], but the ability of sol-gel incorporated iron(III) to form complexes and the following chromogenic reactions of these complexes with antioxidants have not been studied yet.

The aim of the present work was to design new sensor materials for TAC determination by immobilizing the FRAP reagent in the matrix of silica and silica–titania xerogels. The main tasks included: the assembling of the FRAP reagent by immobilizing iron(III) in the xerogels matrix and the following adsorption of 1,10-phenanthroline, the study of the interaction of immobilized FRAP reagent with antioxidants, and the demonstration of analytical application.

## 2. Results and Discussion

The goal of this work was to create sensor materials for TAC determination. The material should change its color when contacting with the antioxidants: iron(III)–Phen complex was chosen as a recognition element for that purpose. We decided to immobilize that complex in sol-gel material by adding iron(III) to the sol mixture, drying the gel, and then modifying the xerogel with 1,10-phenanthroline. TAC determination can be performed using the interaction given in Equation (1). Antioxidant (reducing agent) is denominated as Red, the solid phase is marked by the line above.

(1) $${\bar{\mathrm{Fe}(\mathrm{III})(\mathrm{Phen})}}_{n}+\mathrm{Red}={\bar{\mathrm{Fe}(\mathrm{II})(\mathrm{Phen})}}_{n}+\mathrm{Ox}$$

The sensor material preparation is included the following steps: iron(III) immobilization in the matrix of silica and silica–titania xerogels, the selection of conditions for complex forming reaction between immobilized iron(III) and 1,10-phenathroline, and a material stability study. The main tasks of the sensor material properties study were the following: to assess whether iron(III) ions are retained in the xerogels and keep their complex-forming and oxidation-reduction properties, to study the effect of titanium(IV) and iron(III) content in the xerogel matrix, and to choose the most suitable xerogel and design for the sensor material.

The content of the recognition element in the sensor material—in our case Fe(III)(Phen)_n_ complex (see Equations (1) and (2))—influences the sensitivity of the determination. Usually, the sensitivity is higher when the content of the recognition element in the sensor material is lower. In the present work, the content of the recognition element is defined by the amount of iron(III) immobilized in the sol-gel matrix. We aimed to create silica and silica–titania xerogels with 0.05–1% wt iron(III). Such an amount of iron(III) would be equivalent to 0.2–5 mM iron(III) in the reaction mixture when 0.1 g of xerogel is added to 5.0 mL of sample according to our procedure.

(2) $$\bar{\mathrm{Fe}(\mathrm{III})}+\mathrm{nPhen}+\mathrm{Red}={\bar{\mathrm{Fe}(\mathrm{II})(\mathrm{Phen})}}_{n}+\mathrm{Ox}$$

The present work consisted of the following steps: the synthesis of silica and silica–titania xerogels doped with iron(III), the study of immobilized iron(III) interaction with 1,10-phenanthroline and antioxidants, and the evaluation of the analytical application of the proposed sensor materials.

### 2.1. Silica and Silica–Titania Xerogels Doped with Iron(III) Synthesis

In the present work silica and silica–titania xerogels doped with iron(III) were synthesized by adding iron(III) chloride water solutions to the hydrolyzing mixture. Titanium(IV) tetraethoxyde and tetraethyl orthosilicate were used as precursors. Four different xerogels have been synthesized: silica and silica–titania xerogels doped with 1.0 × 10^−3^ M iron(III) in sol (0, 5.0, and 12.5% vol. of titanium(IV) tetraethoxyde in the precursors’ mixture—SiFe, SiTi5Fe, and SiTi12.5Fe) and silica–titania xerogel doped with 0.1 M iron(III) with 12.5% vol. of titanium(IV) tetraethoxyde in the precursors’ mixture (SiTi12.5Fe100) (Table 1). The wet gels were dried at 800 W microwave irradiation for 10 min. Then, the xerogels were ground and sieved to obtain the fraction of 0.1–0.16 mm particles. Then, xerogels were washed with doubly distilled water and dried again at 800 W microwave irradiation. SiFe, SiTi5Fe, and SiTi12.5Fe were white, and SiTi12.5Fe100 was yellow.

The textural characteristics of the xerogels are very important for the analytical application, so in the present work the influence of titanium(IV) and iron(III) content on the textural characteristics of xerogels doped with iron(III) was investigated (Table 1).

The increase in titanium (IV) content (Table 1, see SiFe, SiTi5Fe, and SiTi12.5Fe) led to a decrease in BET surface area and average pore diameter. The micropore area, on the contrary, increased. The same effect was described earlier for silica–titania xerogels [29,30]. It means that iron(III) in the sol mixture did not substantially influence the gelation process. The increase of iron(III) amount (Table 1, see SiTi12.5Fe and SiTi12.5Fe100) led to a decrease in BET surface area and a small increase of micropores fraction.

**Figure 1:**
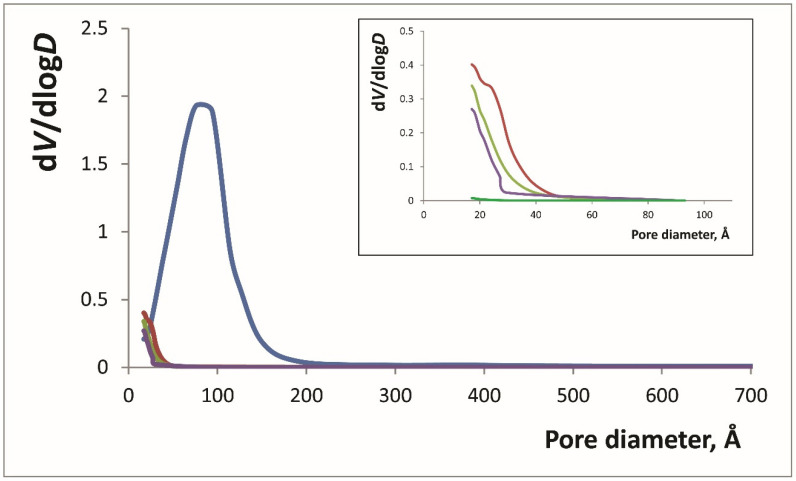
Pore size distribution according to the BJH method calculated from the desorption branches for different xerogels (see Table 1): SiFe (blue), SiTi5Fe (red), SiTi12.5Fe (yellow), SiTi12.5Fe/Phen (purple), SiTi12.5Fe100 (green).

BJH pore distribution analysis of these materials (Figure 1) showed a significant decrease of pore volume of silica–titania xerogels compared to silica xerogel. The increase of iron(III) amount also led to BJH desorption cumulative volume of pores decrease from 0.048 to 0.034 cm^3^/g, mainly focusing on the smallest pores. This effect could probably be explained by the filling of the pores with iron oxide as was described in [23].

We used an EDX analysis for the determination of the iron(III) amount in the synthesized xerogels. For the xerogels with a lower iron(III) content we were unable to detect iron atoms. For SiTi12.5Fe100 xerogel, Si:Ti:Fe ratio in atomic % was measured as 100.0:12.8:5.3 by EDX analysis, while the ratio calculated by the molar amounts added to the sol is 100.0:12.8:7.7. The Si:Ti ratio agrees well with the expected values which we had observed for our silica–titania xerogels before [29]. We believe the difference in the amount of iron can be explained by the fact that EDX analysis measures the elemental composition of the surface of the xerogel particles. Iron atoms are distributed evenly in the sol due to constant mixing, but when the xerogel particles are washed, iron atoms on the surface can be washed off. In this case, the particle surface could contain less iron atoms than expected. For the SiFe, SiTi5Fe, and SiTi12.5Fe xerogels we evaluated iron(III) content by determining the iron(III) concentration in the washing water. The amount of iron(III) in the xerogel was calculated as the difference between the amount loaded into the hydrolyzing mixture and the amount found in the washing water fractions. Better retaining of iron(III) observed for silica–titania xerogels can be explained by their smaller pores. Iron(III) content for all the synthesized xerogels is given below (calculated from washing experiments for SiFe, SiTi5Fe, and SiTi12.5Fe, and calculated from EDX data for SiTi12.5Fe100):
MaterialSiFeSiTi5FeSiTi12.5FeSiTi12.5Fe100Amount of iron(III), % wt0.022 0.032 0.047 2.80

### 2.2. Immobilized Iron(III) Interaction with 1,10-Phenanthroline

To study the applicability of iron(III) doped silica and silica–titania xerogels for TAC determination, we investigated the ability of immobilized iron to form complexes with 1,10-phenathroline and to be reduced by antioxidants with the formation of colored complex (Equation (2)).

**Figure 2:**
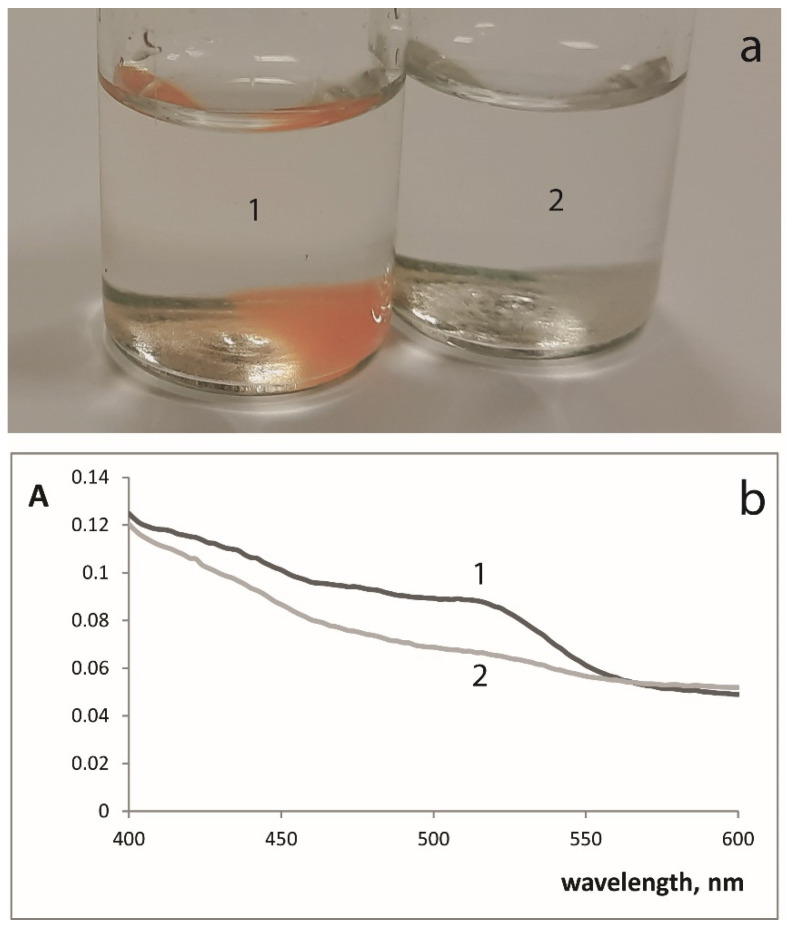
Interaction of SiFe xerogel with 0.075 M 1,10-phenanthroline solution in the presence of 5.0 × 10^−5^ M catechol (1) or in the absence of catechol (2). (**a**) coloration of SiFe xerogel, (**b**) spectra of SiFe xerogel.

The ability of immobilized iron(III) to react with the antioxidants (reducing agents) with the reduction to iron(II) was studied using 1,10-phenanthroline as iron(II) chromogenic ligand and catechol as a model antioxidant. SiFe, SiTi5Fe, and SiTi12.5Fe xerogels changed color from white to red, while the solution remained clear. SiFe xerogel color is given as an example in Figure 2. The spectrum of the xerogels showed a maximum at 510 nm that is characteristic to iron(II)–1,10-phenanthroline complex in solution. No color change was observed for the interaction of SiTi12.5Fe100 with 1,10-phenanthroline and catechol. The increase of iron(III) amount led to the loss of its ability to form complexes with 1,10-phenatroline, probably due to polymerization of iron atoms. SiFe, SiTi5Fe, and SiTi12.5Fe xerogels were chosen for further experiments.

**Figure 3:**
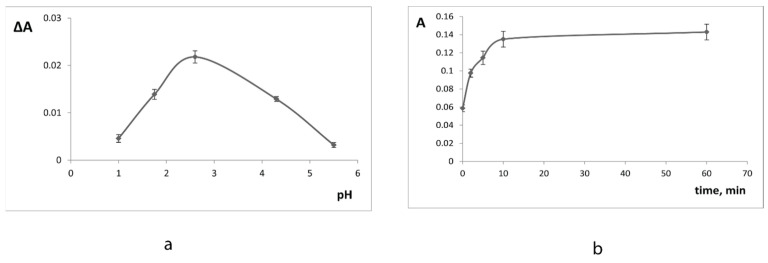
The influence of pH (**a**) and time of contact (**b**) on the interaction of SiFe xerogel with 0.075 M 1,10-phenanthroline and catechol. ΔA = A_catechol_ − A_blank_.

To select the conditions for the modification of the iron(III)-doped xerogels with 1,10-phenanthroline we investigated the influence of pH and time of interaction. We studied the influence of pH in the range of 1.0–5.5 on ΔA value—the difference between the absorbances of xerogels in presence of 0 and 5.0 × 10^−5^ M catechol (Figure 3). Maximal ΔA values were observed at pH 2.0–3.0 and pH 2.6 was chosen for further experiments. The equilibrium was reached at 10 min after the reaction start for SiFe (Figure 3). The reaction was faster for SiTi5Fe and SiTi12.5Fe: the xerogel absorbances did not increase after 5 min of interaction.

Selected conditions were used for sensor materials preparation. Xerogels doped with iron(III) were modified with 1,10-phenanthroline by sorption from the solution and the following air drying. These materials kept their initial white color. Silica and silica–titania xerogels static capacity for 1,10-phenathroline was determined using the interaction described in Equation (3) and equaled 0.207 mmol/g, 0.216 mmol/g, and 0.239 mmol/g for SiFe, SiTi5Fe, and SiTi12.5Fe xerogels, respectively. Static capacity values did not differ significantly for different xerogels and the amount of adsorbed 1,10-phenanthroline exceeded the amount of the immobilized iron(III) (3.9–8.3 μmol/g). That suggests that 1,10-phenanthroline is adsorbed in a non-specific manner.

(3) $$\bar{\mathrm{Fe}(\mathrm{III})}+\mathrm{nPhen}={\bar{\mathrm{Fe}(\mathrm{III})(\mathrm{Phen})}}_{n}$$

The influence of the modification with 1,10-phenanthroline on the textural characteristics of the xerogels was studied for the modified SiTi12.5Fe (SiTi12.5Fe/Phen, Table 1, Figure 1). The observed decrease of surface area and pore volume values can be explained by the adsorption of 1,10-phenanthroline on the xerogel surface. All the parameters, except average pore diameter, decreased by approximately 35% and the average pore diameter did not change; both these facts suggest that 1,10-phenanthroline is adsorbed evenly. This adsorption is similar to the eriochrome cyanine R- Ti(IV) complex formation described earlier [31].

### 2.3. The Analytical Application of the Modified Xerogels

The interaction of all the prepared modified xerogels with antioxidants was studied using catechol as model antioxidant. The time of reaching the equilibrium (when the absorbance of the xerogel stopped increasing) depended on the titanium(IV) content (Table 2). Titanium(IV) incorporated in the matrix of the xerogel accelerated the reduction of iron(III) similarly to heteropoly compounds reduction [29].

The interaction of all the modified xerogels (SiFe/Phen, SiTi5Fe/Phen, and SiTi12.5Fe/Phen) with different antioxidants was studied in the 1.0 × 10^−5^ M–1.0 × 10^−2^ M range and the calibration curve slopes were determined (Table 2). As expected, the increase of titanium(IV) content was accompanied with the increase of the sensitivity (slope value), most probably, due to the increased iron(III) content and the increased rate of its reduction. However, for phenolic compounds this effect was much more pronounced than for sodium sulfite. This can be explained by the formation of the complex between phenolic compounds and titanium (IV) [21], which could result in more efficient extraction of such antioxidants from the solution. These complexes are characterized by the absorbance maximum at 400–420 nm [21], so they are not likely to interfere in this procedure of antioxidants determination. Statistical analysis (t-test) of the data given in Table 2 showed that the slope values increased significantly (*p* < 0.1, n = 3) in almost all cases; the increase of the slope for sulfite was not significant between SiTi5Fe/Phen and SiTi12.5Fe/Phen (*p* = 0.13, n = 3). The differences between two silica–titania xerogels were generally less significant than the differences between silica and silica–titania xerogels.

SiTi12.5Fe/Phen was chosen as a sensor material to develop analytical procedures for the solid phase spectrophotometric antioxidants determination. The absorbance of the xerogels at 510 nm at 5 min was chosen as the analytical signal. The analytical ranges starting from limit of quantitation and limit of detection (LOD) values are given in Table 3.

Immobilization of the iron(III)–Phen complex could affect the input of each antioxidant to TAC value, so it is important to compare the interaction of iron(III)–Phen complex with antioxidants in solution and in solid phase. In order to perform this comparison equivalent antioxidant capacities (EAC) are calculated for both variants using the same standard antioxidant [7,9,10,14]. Gallic acid can be used as such a standard antioxidant, and the resulting EACs are calculated as calibration curve slopes divided by the slope for gallic acid. In [14] the immobilization of iron(III)–Phen complex on Nafion membrane led to two-fold decrease of EAC for ascorbic acid. For the SiTi12.5Fe/Phen procedure, however, EACs for catechol and ascorbic acid differed only by about 10% when compared to the iron(III)–Phen in solution, and these differences were found to be statistically insignificant (Table 4). The difference with Nafion immobilization can be explained by a different 3D structure of the solid phase: small xerogel particles dispersed in water resemble bulk solution phase more than flat and negatively charged Nafion membrane. These results demonstrate that silica–titania xerogel is a suitable and promising matrix for iron(III)–Phen complex immobilization.

The recovery test of the proposed solid phase spectrophotometric TAC determination procedure was performed using a gallic acid standard solution (3.8 × 10^−4^ M). The results were the following: (3.6 ± 0.3) × 10^−4^ M gallic acid was found in this solution (n = 3, *P* = 0.95) and the relative standard deviation equaled 4.3%. The developed analytical procedure was used for TAC determination in tea and soft drink samples (Table 5). No significant differences were found between TAC values obtained by the SiTi12.5Fe/Phen procedure and standard FRAP procedure, which indicates a good agreement. The applicability of the proposed procedure for TAC determination was demonstrated. As SiTi12.5/Phen is an immobilized form of FRAP reagent it can also be applied to other samples TAC determination.

We compared the LOD values of the proposed procedure with other solid phase spectroscopic procedures for antioxidant determination (Table 6). Gallic acid and ascorbic acid determination procedures were chosen for this comparison because they are some of the most popular model antioxidants. LOD values are similar to our previous results [21,29] and comparable with other solid phase spectroscopic procedures. Time of analysis and sensor material stability are important for on-site analytical applications. Time of analysis (Table 6) greatly depends on the matrix type: paper-based matrices allow very fast color development and solid matrices require a rather long time (around 1 h [7,9,10,13,14]). Silica–titania xerogels fit in between these two categories, because on one hand they are solid and, on the other hand, they are highly porous and allow fast analyte diffusion. Stability of the described sensor materials is also given in Table 6. Silica–titania xerogels doped with iron(III) and modified with 1,10-phenanthroline can be stored at room temperature for at least 6 months. These xerogels are based on silica and titania oxides that are considered very safe and inert materials. The amount of immobilized iron is very low (less than 0.1% wt) and the amount of 1,10-phenanthroline is 4 times lower than for the standard FRAP procedure. Based on high stability, safety, and short time of analysis it can be concluded that silica–titania xerogels doped with iron(III) have a great potential for on-site TAC determination.

## 3. Experimental

### 3.1. Reagents

The following reagents were purchased from Acros Organics: catechol, gallic acid, ascorbic acid, sodium sulfite, 1,10-phenanthroline, iron(III) chloride hexahydrate, titanium(IV) tetraethoxyde, and tetraethyl orthosilicate. All the reagents were of analytical grade; titanium(IV) tetraethoxyde was of technical grade.

Stock solutions of catechol, gallic acid, ascorbic acid, and sodium sulfite were prepared with doubly distilled water. Only freshly prepared solutions were used.

### 3.2. Instrumentations

Silica and silica–titania xerogels were obtained by drying in Ethos microwave equipment (Milestone, Italy). Xerogels were obtained by drying in Ethos microwave equipment (Milestone, Italy). Surface area, porosity BET analysis, and BJH pore distribution analysis were carried out with ASAP 2000 (Micromeritics, Norcross, GA, USA). Scanning electron microscopy (SEM) images were collected with the use NVision 40 high-resolution scanning electron microscope (Zeiss, Oberkochen, Germany). Energy-dispersive X-ray analysis (EDX) was performed using X-MAX 80 spectrometer (Oxford Instruments, Abingdon, UK); analysis was performed at 20 kV with 30 μm aperture and the distance to the sample was 4.4 mm.

Absorbance of solutions (l = 1.0 cm) and xerogels water suspensions (l = 0.1 cm) was measured using a Lambda 35 spectrophotometer (PerkinElmer, Waltham, MA, USA) equipped with 50 mm integrating sphere (Labsphere, North Sutton, NH, USA). pH of the reaction mixtures was measured with an HI83303 photometer/pH-meter and HI11310 pH electrode (Hanna Instruments, Woonsocket, RI, USA).

Statistical analysis was carried out using MS Excel. The two-tailed Student test was used for the calculating of *p*-values.

### 3.3. Synthesis of Silica and Silica–Titania Xerogels Doped with Iron(III)

Silica and silica–titania xerogels were obtained using earlier developed procedures [30]: 20.0 mL of 0.05 mol·L^−1^ hydrochloric acid in 50% ethanol solution was added to 10.0 mL of the precursors’ mixture (0, 5.0, or 12.5% vol. titanium(IV) tetraethoxyde) while stirring. To obtain xerogels doped with iron(III) either ferric chloride was added to the sol mixture in order to get the final concentration of 1.0 × 10^−3^ M or 1.0 × 10^−1^ M. The wet gel was formed in the next 72 h. The wet gels were dried at 800 W microwave irradiation for 10 min to get dry xerogels. The xerogels were washed 3 times with 100.0 mL of doubly distilled water and then dried again at 800 W microwave irradiation.

### 3.4. General Procedure to Study the Interaction of Xerogels Doped with Iron(III) Interactions with Antioxidants in Presence of 1,10-Phenanthroline

An amount of 0.10 g of xerogel was added to 4.1 mL of solution, containing 4.0 mL of 1,10-phenanthroline solution at different pH and either 0.1 mL of 2.0 × 10^−3^ M catechol solution or 0.1 mL of distilled water. The obtained mixture was shaken for 1 to 60 min. Then, the xerogels absorbance spectra were recorded. The optimal reaction conditions (pH, time of reaction) were chosen by maximizing the analytical signal (xerogel absorbance) in order to get the maximal sensitivity of future determination procedure.

### 3.5. Preparation of the Sensor Materials—Xerogels Doped with Iron(III) and Modified with 1,10-Phenanthroline

1.0 g of different xerogels was mixed with 40.0 mL of 0.015 M 1,10-phenanthroline solution and shaken for 10 min. Then, the solution was decanted and the residual concentration of 1,10-phenanthroline was measured. This operation was repeated until the residual concentration of 1,10-phenanthroline in the solution stopped decreasing (3 to 5 times). Then, the modified xerogel was dried at room temperature overnight.

### 3.6. General Procedure to Study Interaction of Sensor Material with Antioxidants

An amount of 0.10 g of sensor material was added to 4.0 mL of antioxidant solution (pH 2.6) and the obtained mixture was shaken for 5 min. Then, sensor material absorbance was measured at 510 nm.

### 3.7. Sample Preparation and Solid Phase Spectrophotometric Determination Procedure

An amount of 1.0 g of a tea sample was boiled in 100.0 mL of distilled water. After cooling, the sample was filtered. The filtered tea extract was diluted to the mark of 100.0 mL with distilled water. A soft drink sample was diluted twice with distilled water. pH of the samples was adjusted to 2.6.

TAC determination using sensor material: 0.10 g of sensor material was added to 4.0 mL sample and the obtained mixture was shaken for 5 min. Then, sensor material absorbance was measured at 510 nm and TAC was calculated in gallic acid equivalents using the calibration curve for gallic acid.

TAC determination with Fe(III)–1,10-phenanthroline complex in solution was adapted from [32].

## 4. Conclusions

We have prepared new sol-gel materials—silica and silica–titania xerogels doped with iron(III)—and investigated their properties. The ability of immobilized iron(III) to form complexes with 1,10-phenanthroline and to be reduced to iron(II) has been demonstrated for the first time. The increase of titanium(IV) content has led to a decrease in BET surface area and average pore diameter and the increase of micropore surface area and volume, which has resulted in better iron(III) retention in the xerogels.

Silica and silica–titania xerogels doped with iron(III) have been modified with 1,10-phenanthroline in order to prepare the new sensor materials for FRAP-based antioxidant determination. The immobilization of iron(III)–1,10-phenanthroline complex in xerogel matrix has been performed for the first time. The increase of titanium(IV) content in the sensor material has led to the increase of the reaction rate and the sensitivity of spectrophotometric solid phase determination. Catechol, gallic and ascorbic acids, and sulphite have been used as model antioxidants in order to evaluate the analytical performance of proposed sensor materials. Limits of detection for catechol, gallic and ascorbic acids, and sulphite equal 7.8 × 10^−6^ M, 5.4 × 10^−6^ M, 1.2 × 10^−5^ M, and 3.1 × 10^−4^ M, respectively. Proposed procedures based on new sensor materials have the following advantages: the determination of antioxidants is fast (5 min vs. 30–45 min for other matrices described in literature), and the sensor materials have long-term stability (6 months storage time vs. 15–30 days for other matrices). Using these sensor materials allows a reduction of reagent consumption: the amount of immobilized iron is very low (less than 0.1% wt) and the amount of 1,10-phenanthroline is 4 times lower than for the standard FRAP procedure. Proposed sensor materials have been applied for fast solid phase spectrophotometric determination of total antioxidant capacity (in gallic acid equivalents) in soft beverages.

## Figures and Tables

**Table 1:** Composition and textural characteristics of silica and silica–titania xerogels doped with iron(III).

Material	Composition		Textural Characteristics				
	Titanium(IV) Tetraethoxyde Content, % Vol.	Iron(III) Concentration in Sol, M	BET Surface Area, m^2^/g	Micropore Area, m^2^/g	Total Pore Volume, cm^3^/g	Micropore Volume, cm^3^/g	Average Pore Diameter, Å
SiFe	0	0.001	696	50	0.92	0.01	53.0
SiTi5Fe	5	0.001	567	286	0.27	0.13	19.3
SiTi12.5Fe	12.5	0.001	520	316	0.24	0.14	18.6
SiTi12.5Fe100	12.5	0.1	463	302	0.23	0.15	19.8
SiTi12.5Fe/Phen	12.5	0.001	348	195	0.16	0.09	18.3

**Table 2:** The influence of Ti(VI) content in the modified xerogel matrix on the equilibrium time and sensitivity of antioxidant determination.

Material	Time Required for Interaction with 5.0 × 10^−4^ M Catechol, Min	Slopes, M^−1^		
		Catechol	Gallic Acid	Sodium Sulfite
SiFe/Phen	5	55	193	8
SiTi5Fe/Phen	1	229	855	22
SiTi12.5Fe/Phen	<1	940	1435	24

**Table 3:** Analytical parameters of antioxidants determination using SiTi12.5Fe/Phen.

Analyte	Analytical Range, M	LOD, M (n = 3)
Catechol	2.3 × 10^−5^–1.0 × 10^−3^	7.8 × 10^−6^
Gallic acid	1.6 × 10^−5^–5.0 × 10^−4^	5.4 × 10^−6^
Ascorbic acid	3.5 × 10^−5^–1.0 × 10^−3^	1.2 × 10^−5^
Sodium sulfite	9.4 × 10^−4^–5.0 × 10^−3^	3.1 × 10^−4^

**Table 4:** Gallic acid equivalent antioxidant capacities for the antioxidants determination in solution and using SiTi12.5/Phen.

Analyte	SiTi12.5Fe/Phen	Iron(III)–Phen in Solution	*p*-Value (n = 3)
Catechol	0.65	0.61	0.59
Ascorbic acid	0.43	0.49	0.13
Sodium sulfite	0.02	0.07	0.006

**Table 5:** Total antioxidant capacity determination in beverages using SiTi12.5/Phen as sensor material (n = 3, *P* = 0.95).

Sample	TAC, M (GA Equivalents)		
	SiTi12.5Fe/Phen	Iron(III)–Phen in Solution	*p*-Value
Black tea	(1.59 ± 0.17) × 10^−4^	(1.50 ± 0.10) × 10^−4^	0.36
Juice containing soft drink	(1.16 ± 0.08) × 10^−4^	(1.19 ± 0.02) × 10^−4^	0.38

**Table 6:** Comparison for solid phase spectroscopic procedures for TAC determination.

Sensor Material	Analytical Signal	LOD, M		Time of Analysis, Min	Storage Time	Reference
		Gallic Acid	Ascorbic Acid			
Iron(III) and 1,10-phenanthroline immobilized in polymethacrylate matrix	Sensor material absorbance	5.8 × 10^−6^	2.8 × 10^−5^	45	Not studied	[13]
Iron(III) and 1,10-phenanthroline immobilized on Nafion membrane	Sensor material absorbance	4.6 × 10^−7^	4.4 × 10^−6^	30	30 days	[14]
Copper(II)–neocuproine immobilized on carrageenan film	Sensor material absorbance	2.3 × 10^−6^	3.6 × 10^−6^	90	14 days	[7]
Copper(II)–neocuproine immobilized on Nafion membrane	Sensor material absorbance	3.1 × 10^−7^	2.1 × 10^−6^	30	15 days	[8]
	Sensor material reflectance	3.0 × 10^−7^	1.2 × 10^−6^	30	15 days	[9]
Iron(III) immobilized on paper with multilayers of surfactants	Sensor material color	3.5 × 10^−7^	-	Immediate	Not studied	[2]
Cerium(IV) nanoparticles immobilized on paper	Length of the colored zone of the sensor material	5.0 × 10^−6^	8.0 × 10^−6^	Time required for paper drying	50 days	[16]
Silica–titania xerogel	Sensor material absorbance	5.9 × 10^−6^	1.1 × 10^−5^	7–10	12 months	[21]
Silica–titania xerogel doped with Mo,P-heteropoly compounds	Sensor material absorbance	-	4.0 × 10^−6^	20	6 months	[29]
Silica–titania xerogel doped with iron(III) and modified with 1,10-phenanthroline	Sensor material absorbance	5.4 × 10^−6^	1.2 × 10^−5^	5	6 months	Present work

## Data Availability

Data sharing is not applicable to this article.
